# Metastatic patterns and prognosis of patients with primary malignant cardiac tumor

**DOI:** 10.3389/fcvm.2022.1009765

**Published:** 2022-12-05

**Authors:** Tianwang Guan, Qingqian Wei, Yongshi Tang, Hongjun Zhao, Zhenxing Lu, Weijing Feng, Yintong Teng, Zehao Luo, Kaiyi Chi, Caiwen Ou, Minsheng Chen

**Affiliations:** ^1^Department of Cardiology, Laboratory of Heart Center, Zhujiang Hospital, Southern Medical University, Guangzhou, China; ^2^Guangdong Provincial Biomedical Engineering Technology Research Center for Cardiovascular Disease, Sino-Japanese Cooperation Platform for Translational Research in Heart Failure, Guangzhou, China; ^3^Department of Clinical Medicine, Clinical Medical School, Guangzhou Medical University, Guangzhou, China; ^4^State Key Laboratory of Organ Failure Research, Guangdong Provincial Key Lab of Shock and Microcirculation, Department of Cardiology, Nanfang Hospital, Southern Medical University, Guangzhou, China; ^5^Dongguan Hospital of Southern Medical University, Southern Medical University, Dongguan, China

**Keywords:** distant metastasis, prognosis, primary malignant cardiac tumor, Surveillance, Epidemiology, and End Results (SEER) database, cardio-oncology

## Abstract

**Background:**

Distant metastases are independent negative prognostic factors for patients with primary malignant cardiac tumors (PMCT). This study aims to further investigate metastatic patterns and their prognostic effects in patients with PMCT.

**Materials and methods:**

This multicenter retrospective study included 218 patients with PMCT diagnosed between 2010 and 2017 from Surveillance, Epidemiology, and End Results (SEER) database. Logistic regression was utilized to identify metastatic risk factors. A Chi-square test was performed to assess the metastatic rate. Kaplan–Meier methods and Cox regression analysis were used to analyze the prognostic effects of metastatic patterns.

**Results:**

Sarcoma (*p* = 0.002) and tumor size¿4 cm (*p* = 0.006) were independent risk factors of distant metastases in patients with PMCT. Single lung metastasis (about 34%) was the most common of all metastatic patterns, and lung metastases occurred more frequently (17.9%) than bone, liver, and brain. Brain metastases had worst overall survival (OS) and cancer-specific survival (CSS) among other metastases, like lung, bone, liver, and brain (OS: HR = 3.20, 95% CI: 1.02–10.00, *p* = 0.046; CSS: HR = 3.53, 95% CI: 1.09–11.47, *p* = 0.036).

**Conclusion:**

Patients with PMCT who had sarcoma or a tumor larger than 4 cm had a higher risk of distant metastases. Lung was the most common metastatic site, and brain metastases had worst survival among others, such as lung, bone, liver, and brain. The results of this study provide insight for early detection, diagnosis, and treatment of distant metastases associated with PMCT.

## Introduction

Primary malignant cardiac tumors (PMCTs) are rare but deadly carcinomas that originate from the heart in a highly aggressive manner (1, 2). Despite improvements in treatment strategies, PMCT prognosis remains disappointing, with 5-year survival rates of 11.5% (1). The high rate of metastases limits the treatment efficiency. Many patients with PMCT have limited options for curative measures due to metastases and suffer from consequent metastatic complications that might even result in death (3, 4). Previous studies identified distant metastases as a prognostic risk factor for PMCT (5, 6), thus emphasizing the need for further exploration of metastatic patterns and their related prognostic outcomes.

Most studies have focused on the characteristics and survival of patients suffering from PMCT (1, 2, 7–9). Although some studies have highlighted the adverse prognostic effects of distant metastases in PMCT and primary cardiac sarcomas (4–6, 10), none of the studies have explored the metastatic rate and distribution of metastases, not to mention the prognostic significance of different metastases patterns in PMCT. Thus, there is a lack of thorough and systematic analysis of metastases patterns in PMCT.

Since PMCT is rare, a large randomized controlled trial is not feasible (2). Surveillance, Epidemiology, and End Results (SEER) is an authoritative database on cancer, which contains data from 18 registries in US (11). It can fulfill the deficiency of limited cases reported to a single registry and the significant missing data in case reports. This multicenter retrospective study aimed to evaluate the different metastatic patterns and their prognostic effects in patients with PMCT, offering better insights and providing a scientific basis for improving the prognosis of these patients.

## Patients and methods

### Data resources

This multicenter study used the SEER database, a US national dataset commonly used in cancer and cardio-oncology research. The data collection procedures were standardized to prevent monitoring deviations in SEER (12). As the data is publicly available, ethical approval was unnecessary for this study (13).

### Patient selection

In this study, the inclusion criteria included: (a) case selection (site and morphology, primary site labeled) = “C38.0-Heart”; (b) pathological diagnosis between 2010 and 2017; (c) cases with active follow-up information. The exclusion criteria included: (a) cases with multiple primary tumors; (b) unknown metastatic status; (c) diagnosed only by autopsy or death certificate.

### Variable classification and outcomes

The following SEER variables were collected: distant metastases (yes/no), age at diagnosis (≤ 65 years, > 65 years) (14), sex (male/female), median household income (< 35,000 dollars, 35,000—49,999 dollars, 50,000—74,999 dollars, ≥ 75,000 dollars), year of diagnosis (2010—2013, 2014—2017), race (White, Black, and others), histopathology (lymphomas, sarcomas, mesotheliomas, and others) (2, 5), grade (low, high, others, or unknown) (5), tumor size (≤ 4 cm, > 4 cm) (6), and surgery (yes, no, or unknown). Due to the lack of data on comorbidities, radiation therapy, and chemotherapy in SEER, we didn’t include these variables as the previous studies (15, 16). Distant metastases were defined in the SEER database as the state of metastases to the distant organs at the time of the first diagnosis of PMCT (17). Metastatic sites included the liver, lung, bone, and brain since the SEER database only recorded these common metastatic sites from 2010 (18). Overall survival (OS) refers to the time from the date of the first diagnosis of PMCT to the date of death or the last follow-up. Cancer-specific survival (CSS) was the survival time from primary diagnosis to the death caused by cancer. The follow-up time was defined as the time from the date of the first diagnosis with PMCT to the final follow-up or death. The last follow-up date for this study was December 31, 2017.

### Statistical analysis

The statistical analysis was performed on SPSS version 25.0 (SPSS, Chicago, IL, USA) and R software version 3.6.1.^1^ The categorical variables in the baseline characteristics of patients were calculated using the Chi-square test (19). The metastatic rate of clinical factors was compared by the Chi-square test. The risk factors for distant metastases in patients with PMCT were determined by univariable and multivariable logistic regression analysis. Multivariate logistic regression analyses adjusted statistically significant factors according to univariate analysis (histopathology and tumor size). Kaplan–Meier method (log-rank test), univariate and multivariate Cox regression analyses (enter method) were performed to analyze OS and CSS (20). Among multivariate Cox regression analyses, all variables in univariate analysis with *p*-value < 0.05 were entered into model 1 (age at diagnosis, median household income, histopathology and size); Model 2: it is the same as Model 1, and also including sex. Sex is one of the important sociodemographic characteristics in cardiovascular disease; Model 3: it is the same as Model 2, and also including surgery (21–23). Surgical resection is one of the important treatments of PMCT. In the final model, *p*-value of less than 0.05 was used as a base to identify factors having a statistically significant association with OS and CSS. *p*-value < 0.05 was considered statistically significant, and the confidence interval (CI) was 95%.

## Results

### Patient characteristics

As shown in Table 1, 218 patients with PMCT were included in this study, of which 82 (37.6%) were diagnosed with distant metastases, 147 (67.4%) were less than 65 years at diagnosis, 110 (50.5%) were male, 162 (74.3%) were White, 96 (44.0%) were high grade, 145 (66.5%) had sarcoma, 195 (89.4%) were not a paired, and 100 (45.9%) had large size tumors (> 4 cm). Higher percentage of metastases was observed among patients with large size tumors (> 4 cm) compared to those with smaller one (≤ 4 cm) (43.0% vs. 8.7%, *p* = 0.008). The other clinical variables did not show significant statistical differences (*p* > 0.05). The mean follow-up duration was 3.64 years (SD 0.3 years) for 218 patients with PMCT.

**TABLE 1:** Baseline characteristics of primary malignant cardiac tumors.

		Distant metastasis n (%)		
		
Variable	Total n (%)	No	Yes	*P* value
Total	218 (100.0)	136 (62.4)	82 (37.6)	
Age at diagnosis				0.18
≤ 65 years	147 (67.4)	87 (59.2)	60 (40.8)	
> 65 years	71 (32.6)	49 (69.0)	22 (31.0)	
Median household income				0.46
US < $35,000	0 (0.0)	0 (0.0)	0 (0.0)	
US $35,000-$49,999	32 (14.7)	17 (53.1)	15 (46.9)	
US $50,000-$74,999	127 (58.3)	80 (63.0)	47 (37.0)	
≥ US $75,000	59 (27.1)	39 (66.1)	20 (33.9)	
Sex				0.33
Male	110 (50.5)	65 (59.1)	45 (40.9)	
Female	108 (49.5)	71 (65.7)	37 (34.3)	
Year of diagnosis				0.13
2010–2013	102 (46.8)	58 (56.9)	44 (43.1)	
2014–2017	116 (53.2)	78 (67.2)	38 (32.8)	
Race				0.21
White	162 (74.3)	105 (64.8)	57 (35.2)	
Black	28 (12.8)	18 (64.3)	10 (35.7)	
Others*	28 (12.8)	13 (46.4)	15 (53.6)	
Grade				0.11
Low	7 (3.2)	6 (85.7)	1 (14.1)	
High	96 (44.0)	58 (60.4)	38 (39.6)	
Others^†^	57 (26.1)	41 (71.9)	16 (28.1)	
Unknown	58 (26.6)	31 (53.4)	27 (46.6)	
Histopathology				0.090
Lymphoma	59 (27.1)	43 (72.9)	16 (27.1)	
Sarcoma	145 (66.5)	83 (57.2)	62 (42.8)	
Mesothelioma and others	14 (6.4)	10 (71.4)	4 (28.6)	
Size				0.008
≤ 4 cm	23 (10.6)	21 (91.3)	2 (8.7)	
> 4 cm	100 (45.9)	57 (57.0)	43 (43.0)	
Unknown	95 (43.6)	58 (61.1)	37 (38.9)	
Laterality				0.682
Right	10 (4.6)	6 (4.4)	4 (4.9)	
Left	12 (5.5)	9 (6.6)	3 (3.7)	
Not a paired site	195 (89.4)	120 (88.2)	75 (91.5)	
Bilateral	1 (0.7)	0	1 (0.5)	
Surgery				0.089
No	103 (47.2)	59 (57.3)	44 (42.7)	
Yes	114 (52.3)	77 (67.5)	37 (32.5)	
Unknown	1 (0.5)	0 (0.0)	1 (100.0)	

*Other races include American Indian/Alaska Native, Asian/Pacific Islander.

^†^Others include B-cell, pre-B, B-precursor, and B-cell.

### Factors related to distant metastases

In the univariate logistic regression analysis, sarcoma (*p* = 0.039) and size > 4 cm (*p* = 0.007) were related to metastases in patients with PMCT. The multivariate logistic regression analysis further showed that compared with lymphoma, sarcoma had a higher risk of distant metastases (OR: 3.72, 95% Cl: 1.59–8.70, *p* = 0.002), while mesothelioma and others revealed no statistically significant risk (*p* > 0.05). The risk of distant metastases in larger tumor sizes > 4 cm was higher than in smaller ones ≤ 4 cm (OR: 8.43, 95% CI: 1.87–38.02, *p* = 0.006). Other variables did not demonstrate a statistically significant difference (*p* > 0.05) (Table 2).

**TABLE 2:** Logistic regression analyses of the risk factors for metastasis.

	Univariate analysis		Multivariate analysis^#^	
		
Variable	OR (95% CI)	*P* value	OR (95% CI)	*P* value
Age at diagnosis				
≤ 65 years	Reference		–	–
> 65 years	0.65 (0.36–1.19)	0.16	–	–
Median household income		0.47		
US $35,000-$49,999	Reference		–	–
US $50,000-$74,999	0.67 (0.31–1.46)	0.31	–	–
≥ US $75,000	0.58 (0.24–1.40)	0.23	–	–
Sex				
Male	Reference		–	–
Female	0.75 (0.43–1.31)	0.31	–	–
Year of diagnosis				
2010–2013	Reference		–	–
2014–2017	0.64 (0.37–1.11)	0.12	–	–
Race		0.18		
White	Reference		–	–
Black	1.02 (0.44–2.37)	0.96	–	–
Others*	2.13 (0.95–4.78)	0.068	–	–
Grade		0.13		
Low	Reference		–	–
High	3.93 (0.46–33.96)	0.21	–	–
Others_†_	2.34 (0.26–21.01)	0.45	–	–
Unknown	5.23 (0.59–46.18)	0.14	–	–
Histopathology		0.090		0.007
Lymphoma	Reference		Reference	
Sarcoma	2.01 (1.04–3.89)	0.039	3.72 (1.59–8.70)	0.002
Mesothelioma and others	1.08 (0.30–3.92)	0.91	1.52 (0.39–5.89)	0.55
Size		0.026		0.004
≤ 4 cm	Reference		Reference	
> 4 cm	7.92 (1.76–35.62)	0.007	8.43 (1.87–38.02)	0.006
Unknown	6.70 (1.48–30.26)	0.013	14.48 (2.96–70.84)	0.001

^#^Multivariate logistic regression analyses adjusted statistically significant factors according to univariate analysis (histopathology and tumor size).

*Other races include American Indian/Alaska Native, Asian/Pacific Islander.

^†^Others include B-cell, pre-B, B-precursor, and B-cell.

OR, odds ratio; CI, confidence interval.

### Incidence of distant metastases

**FIGURE 1:**
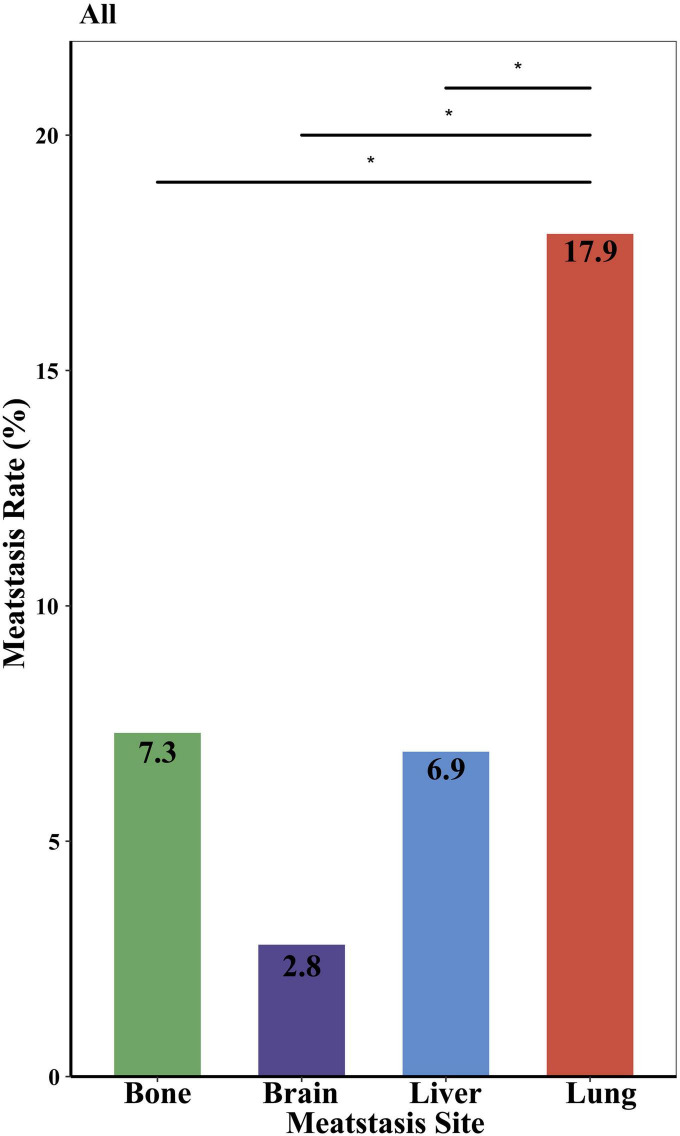
Incidence of distant metastases in different metastatic sites. *P < 0.05.

As shown in Figure 1, the incidence of lung metastases was the highest (17.9%), followed by bone and liver (7.3 and 6.9%, respectively) (*p* < 0.05). Brain metastases were the rarest, detected in 2.8% of patients with distant metastases. As for the age at diagnosis, sex, race, tumor grade, year of diagnosis, histopathology, tumor size, surgery, and median household income subgroups, the same trend was also found in these subgroups. The most common site of occurring metastases was the lung, and the least site was the brain (Supplementary Figure 1). It was also observed that Black PMCT, patients with sarcomas, tumor size ¿ 4 cm, no surgery, and low household income subgroups developed lung metastases more frequently (about 25%) (Supplementary Figure 1). As shown in Supplementary Figure 2, tumors originated in the right-hearted site show a noteworthy higher proportion of lung metastases, at 50%, and higher incidence of lung metastases, at 30% (*p* > 0.05). In left sited heart, the proportion and incidence of brain metastases are 0 (Supplementary Figure 2).

### The proportion of distant metastases

**FIGURE 2:**
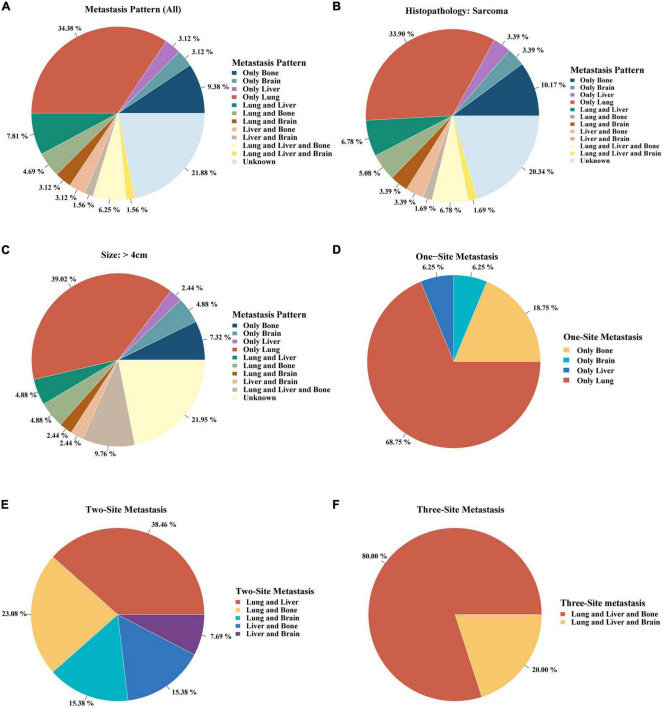
The proportion of distant metastases in different metastatic patterns.

Single lung metastasis (34.38%) was the most frequent site of all metastatic patterns (Figure 2A). Single site metastasis, approximately 50%, was the most common pattern compared to two- and three-site metastases (Figure 2A). As sarcoma and tumor size > 4 cm were independent risk factors of distant metastases, we further investigated the metastatic pattern in the sarcoma and large tumor size > 4 cm subgroups. As shown in Figures 2B,C, single lung metastasis was also the primary metastatic pattern in both sarcoma and large tumor size (> 4 cm) (33.90 and 39.02%, respectively). Single metastasis occurred most frequently in the lungs (68.75%) (Figure 2D). Lung and liver metastases were the most common pattern of two-site metastases (38.46%) (Figure 2E). Three-site metastases occurred most frequently in the lung, liver, and bone (80%) (Figure 2F).

### The prognostic impacts of distant metastases

**FIGURE 3:**
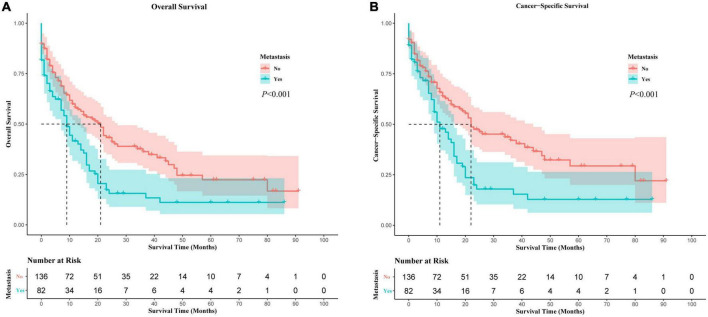
The prognostic impacts of distant metastases.

PMCT was the leading cause of death for all these patients, including distant metastases or non-metastases (Supplementary Figure 3). As PMCT accounts for the most proportion of deaths, we further investigated its prognostic factors related to OS and CSS. In Kaplan-Meier survival analysis, distant metastases were associated with worse OS and CSS compared to PMCT patients without metastases (*p* < 0.05) (Figure 3). Further multivariate analysis showed that distant metastases were an independent prognostic risk factor of OS and CSS (OS: HR = 1.47, 95% CI: 1.05–2.066, *p* = 0.025; CCS: HR = 1.473, 95% CI: 1.02–2.127, *p* = 0.039) (Supplementary Tables 1, 2).

### The prognostic impacts of metastases site

In Kaplan-Meier (Supplementary Figure 4) and univariate Cox analyses (Table 3), the prognostic difference in PMCT patients among metastatic sites of brain, lung, bone, and liver were statistically insignificant (*p* > 0.05). After adjusting the confounding factors (model 1), the multivariate analysis found that brain metastases had the worst OS and CSS among the four sites (in model 1, OS: HR = 3.20, 95% CI: 1.02–10.00, *p* = 0.046; CSS: HR = 3.53, 95% CI: 1.09–11.47, *p* = 0.036) (Table 3). After further adjustment (model 2 and 3), adjusted HR did not change distinctively, and brain metastases still had the worst OS and CSS among the four sites (in model 2, OS: HR = 3.23, 95% CI: 1.01–10.28, *p* = 0.047; CSS: HR = 3.53, 95% CI: 1.07–11.69, *p* = 0.039; in model 3, OS: HR = 3.30, 95% CI: 1.03–10.60, *p* = 0.045; CSS: HR = 3.52, 95% CI: 1.05–11.75, *p* = 0.041) (Table 3).

**TABLE 3:** The prognostic impacts of metastatic sites on patients with primary malignant cardiac tumors.

	OS		CSS	
		
Variable	HR (95% CI)	*P* value	HR (95% CI)	*P* value
**Unadjusted HR**				
Metastasis site		0.75		0.63
Bone	Reference		Reference	
Brain	1.72 (0.62–4.77)	0.29	1.96 (0.69–5.53)	0.21
Liver	1.12 (0.47–2.70)	0.79	1.13 (0.45–2.86)	0.79
Lung	1.26 (0.62–2.58)	0.53	1.27 (0.60–2.70)	0.54
Model 1*				
Metastasis site		0.24		0.19
Bone	Reference		Reference	
Brain	3.20 (1.02–10.00)	0.046	3.53 (1.09–11.47)	0.036
Liver	1.34 (0.53–3.42)	0.53	1.30 (0.49–3.45)	0.60
Lung	1.67 (0.75–3.71)	0.21	1.60 (0.69–3.72)	0.27
**Model 2**_†_				
Metastasis site		0.24		0.20
Bone	Reference		Reference	
Brain	3.23 (1.01–10.28)	0.047	3.53 (1.07–11.69)	0.039
Liver	1.36 (0.52–3.56)	0.53	1.30 (0.47–3.56)	0.62
Lung	1.69 (0.74–3.87)	0.21	1.60 (0.67–3.83)	0.29
**Model 3^‡^**				
Metastasis site		0.23		0.21
Bone	Reference		Reference	
Brain	3.30 (1.03–10.60)	0.045	3.52 (1.05–11.75)	0.041
Liver	1.37 (0.52–3.57)	0.52	1.30 (0.47–3.56)	0.62
Lung	1.67 (0.73–3.82)	0.23	1.60 (0.67–3.85)	0.29

*HR were adjusted for age at diagnosis, median household income, histopathology, and size.

^†^HR were adjusted for age at diagnosis, median household income, histopathology, size, and sex.

^‡^HR were adjusted forage at diagnosis, median household income, histopathology, size, sex, and surgery.

OS, overall survival; CSS, cancer specific survival; HR, hazard ratio; CI, confidence interval.

## Discussion

Using multicenter cohort data, we conducted the largest comprehensive study on different metastatic patterns of patients with PMCT and their prognostic effects. Moreover, we found that histopathology confirmed sarcoma and tumor size > 4 cm associated with a higher risk of distant metastases. The proportion of single lung metastasis (about 34%) was the highest among all metastatic patterns, and the incidence of lung metastases was also the highest (17.9%) among the lung, bone, liver, and brain metastases. The presence of distant metastases negatively impacted the prognosis of patients with PMCT, among which brain metastases was the most fatal among all four.

A higher risk of distant metastases was observed in cardiac sarcoma patients with PMCT compared to cardiac lymphoma, which both univariate and multivariate analyses have confirmed. Patients with cardiac sarcoma were about 3.7 times more likely to develop distant metastases than those with cardiac lymphoma. There was no previous study about the risk factor of distant metastases in PMCT, but similar results were reported in other cancers (24–26). A large cohort study found that different histopathology was related to the different metastatic rates in lung cancer patients (24). Previous studies indicated that soft-tissue sarcomas were prone to metastasizing (27, 28). The other metastatic risk factor was a larger tumor size > 4 cm, associated with a higher risk of distant metastases than a smaller tumor size ≤ 4 cm in patients with PMCT. This finding was in line with previous SEER-based studies, which showed that the risk of metastases increases with the increased tumor thickness in thyroid cancer (25) and inflammatory breast cancer (26). These findings may help clinicians identify the patients with PMCT at high risk of metastases.

We found that up to 37.6% of patients with PMCT had distant metastases at the time of diagnosis. Our results were in line with the previous study finding that the incidence of distant metastases was 38.7% in PMCT (5). In comparison, only 3.0–7.8% of breast cancer patients had distant metastases at the time of diagnosis due to the improvements in earlier diagnosis (16, 17). The high rate of distant metastases in PMCT may be due to the inadequate understanding of metastatic patterns and overdue screening because of its rarity (1, 2). Most clinicians encounter very few cases of PMCT during their practice (29), and its non-specific symptoms make early diagnosis challenging (4). For the efficiency of treatment always limited by distant metastases, it was emphasized that both cardiologists and cardiac surgeons should be aware that a PMCT which has not metastasized yet should be treated in time (29), thus preventing distant metastases and a worse prognosis.

We reported for the first time that single lung metastasis (about 34%) was the most common of all metastatic patterns and the incidence of lung metastases (17.9%) was the highest of all lungs, bone, liver, and brain metastases. Notably, the rate of lung metastases in PMCT (17.9%) was significantly higher than the overall rate (4.04%) of lung metastases in 29 cancer sites (30). The rate of lung metastases in PMCT was also higher than in patients with lung and bronchus cancer (12.41—13.74%) (30). Patients with PMCT with specific characteristics, i.e., Black, sarcoma, tumor size¿4 cm, no history of surgery, and low household income subgroups, developed lung metastases more frequently, up to about 25%. Previous studies observed comparable results demonstrating that the lung was the common metastatic site in primary cardiac sarcoma (31). Our results of laterality echo the results of histopathology subgroups. Angiosarcoma is the most common primary cardiac sarcoma, which is predominantly located in the right heart (32). Right heart sarcomas were found to be more infiltrative and metastasize earlier than left-sited and pericardiac ones (33). These findings may be due to the pulmonary circulation originating from right ventricle, which contributes to the metastases of cancer cells from the right heart to the lung, and primary cardiac sarcoma originates primarily from the right atrium (34). Nevertheless, we also found that brain metastases were the rarest (about 2.8%) of the four sites in PMCT patients, slightly higher than the average rate (2.0%) of brain metastases in cancer patients (35). Previous studies have shown the tumor in left sited heart tend to have a bigger chance of brain metastases (36). Due to the small case of right and left cardiac tumor in SEER, further studies are need to explore the association between tumor laterality and distant metastasis. Brain metastases commonly occur in melanoma, breast cancer, and lung cancer patients (35, 37) reflecting the heterogeneity of distant metastases in various cancers. It is also worth mentioning the impact of household income on incidence of lung metastases. In line with our results, previous study found that socioeconomic status, such as insurance status, were associated with survival in patients with cardiac diffuse large B-cell lymphoma (38). The limitation of access to early detection, early diagnosis and early treatment caused by the low-income situation may explain the high incidence of lung metastases at diagnosis (38–40). Therefore, clinical physicians should pay more attention to screening and detecting distant metastases, particularly lung metastases, and make better diagnosis and treatment strategies for PMCT patients with lung metastases.

Previous studies showed that distant metastases were associated with worse OS and CSS in PMCT, but the prognostic effects of different metastatic patterns were not explored (5, 6, 10). Our study revealed that brain metastases were the most lethal among the four metastases sites in PMCT. Patients with PMCT with brain metastases had a higher risk of all-cause death and cancer-specific death, approximately 3.2 and 3.5 times higher than patients with PMCT with bone metastases, respectively. Although the hazard ratio (HR) for the mortality risk of brain metastases had a wide confidence interval, the adjusted HRs did not change distinctively in sensitive analysis (model 1, 2, and 3). The wide confidence intervals of HR could be attributed to the limited cases of single-site metastases. A large-scale study is needed to verify these findings. The results of our study were in line with one of the population-based studies that found that liver cancer patients with brain metastases had the worst survival rates compared to patients with lung, bone, or liver metastases (41). A retrospective cohort study also reported that brain-only metastasis had the worst survival among brain-, lung-, bone-, liver-only, and multiple-site metastases (42). It is generally related to poor survival and poses significant clinical challenges (35) because the blood-brain barrier has limited the use of many systemic chemotherapeutic agents in treatment (43).

### Limitation

There were some limitations in this study. First, data on distant solid organ metastases was collected in 2010, including the common sites: bone, brain, liver, and lung in the SEER database (17). Therefore, we could not explore distant metastases of other sites in PMCT. Nevertheless, these five metastatic sites in our study accounted for a large proportion (more than 87%) of all metastatic patients, and a few patients with distant metastases in other sites were missing. Furthermore, even with the multicenter data, we had a limited number of patients in specific metastatic pattern categories, as reflected in the wide confidence intervals of HR. Although the date of distant metastases was available at the time of PMCT diagnosis, the SEER database did not provide information about recurrence, and we can’t further study the metastatic pattern after recurrence. Nevertheless, a significant strength of our study was the long-term follow-up and multicenter date (18 registries in the US). Although previous studies have reported the adverse prognostic effects of distant metastases in PMCT (4–6, 10), to the best of our knowledge, this is the first and largest comprehensive analysis of metastatic patterns of patients with PMCT and their prognostic effects.

## Conclusion

Over a third of patients with PMCT had distant metastases at the time of diagnosis. Sarcoma and tumor size¿4 cm were associated with a greater risk of distant metastases in patients with PMCT. Among the lung, bone, liver, and brain, lung metastases were the most common, and brain metastases had the worst OS and CSS. This study contributed to a better understanding of metastatic patterns and their prognostic effects in patients with PMCT. The prognosis of patients with PMCT can be improved by adopting strategies for early detection, diagnosis, and treatment of distant metastases.

## Data availability statement

Publicly available datasets were analyzed in this study. This data can be found here: The data in our study are available from the SEER https://seer.cancer.gov.

## Ethics statement

Ethical review and approval was not required for the publicly available data in this study.

## Author contributions

TG: conception, data collection, study design, analysis and methodology, interpretation of date, figures design, writing—original draft, and writing—review and editing. QW: study design, analysis and methodology, interpretation of date, figures design, writing—original draft, and writing—review and editing. YsT and HZ: analysis and methodology, interpretation of date, figures design, and writing—original draft. ZxL, WF, and YtT: analysis, interpretation of date, and writing—original draft. ZhL and KC: study design, interpretation of date, and writing—review and editing. CO and MC: funding acquisition, project administration and supervision, and article—review and editing. All authors contributed to the article and approved the submitted version.

## Footnotes

1
https://www.r-project.org

## Abbreviations

CI: confidence intervals
CSS: cancer-specific survival
HR: hazard ratio
OS: overall survival
PMCT: primary malignant cardiac tumor
SEER: Surveillance Epidemiology and End Results.
