# Supplementary material for: Metastatic patterns and prognosis of patients with primary malignant cardiac tumor
Source: Front Cardiovasc Med. 2022 Dec 5;9:1009765. doi: 10.3389/fcvm.2022.1009765 (PMC9760733; doi:10.3389/fcvm.2022.1009765)
Supplement: Supplementary file 1 [file Data_Sheet_1.docx]

**Supplement Material**

**Supplementary Figure legends**

**Supplementary Figure S1.** Incidence of Distant Metastasis of Different Metastatic Sites in subgroups.

**P*＜0.05, NS= Not Significant.

**Supplementary Figure S2.** Proportion and incidence of Distant Metastasis of Different Metastatic Sites in Laterality subgroups. A-C: the proportion of distant metastases; D-F: the incidence of distant metastases.

**Supplementary Figure S3.** The Proportion of Death in Patients with Primary Malignant Cardiac Tumors

DM= Distant Metastasis

**Supplementary Figure S4.** The Prognostic Impacts of Metastasis Site.

**Supplementary Table legends**

**Supplementary Table S1.** Cox Regression Analyses of Prognostic Factors for OS in Primary Malignant Cardiac Tumors

*Others races include American Indian/Alaska Native, Asian/Pacific Islander.

†Others includes B-cell, pre-B, B-precursor and B-cell.

OS = overall survival; HR =hazard ratio; CI = confidence interval.

**Supplementary Table S2.** Cox Regression Analyses of Prognostic Factors for CSS in Primary Malignant Cardiac Tumors

*Others races include American Indian/Alaska Native, Asian/Pacific Islander.

†Others includes B-cell, pre-B, B-precursor and B-cell.

OS = overall survival; HR =hazard ratio; CI = confidence interval.


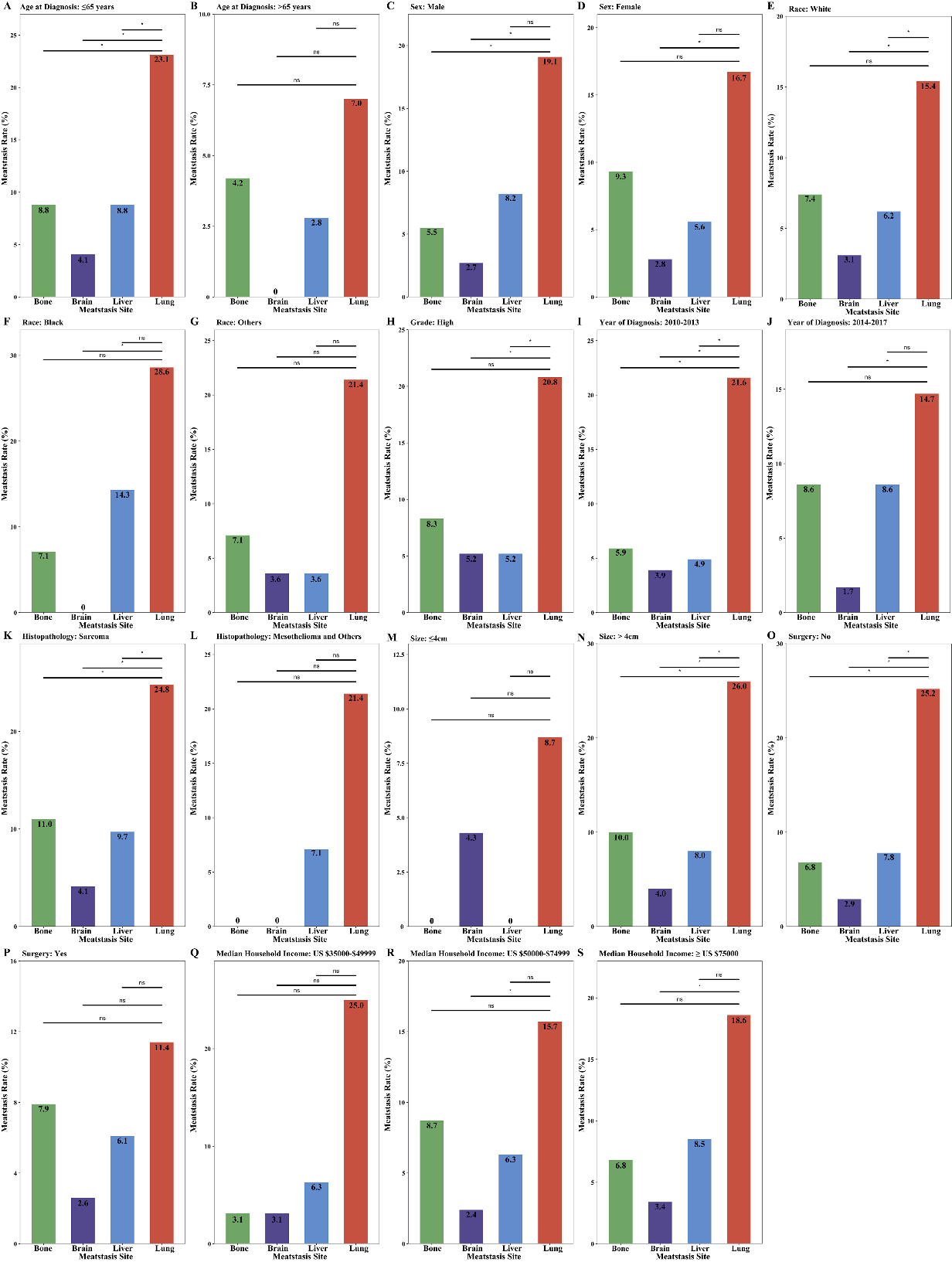


**Supplementary Figure S1.** Incidence of Distant Metastasis of Different Metastatic Sites in subgroups.

**P*＜0.05, NS= Not Significant.


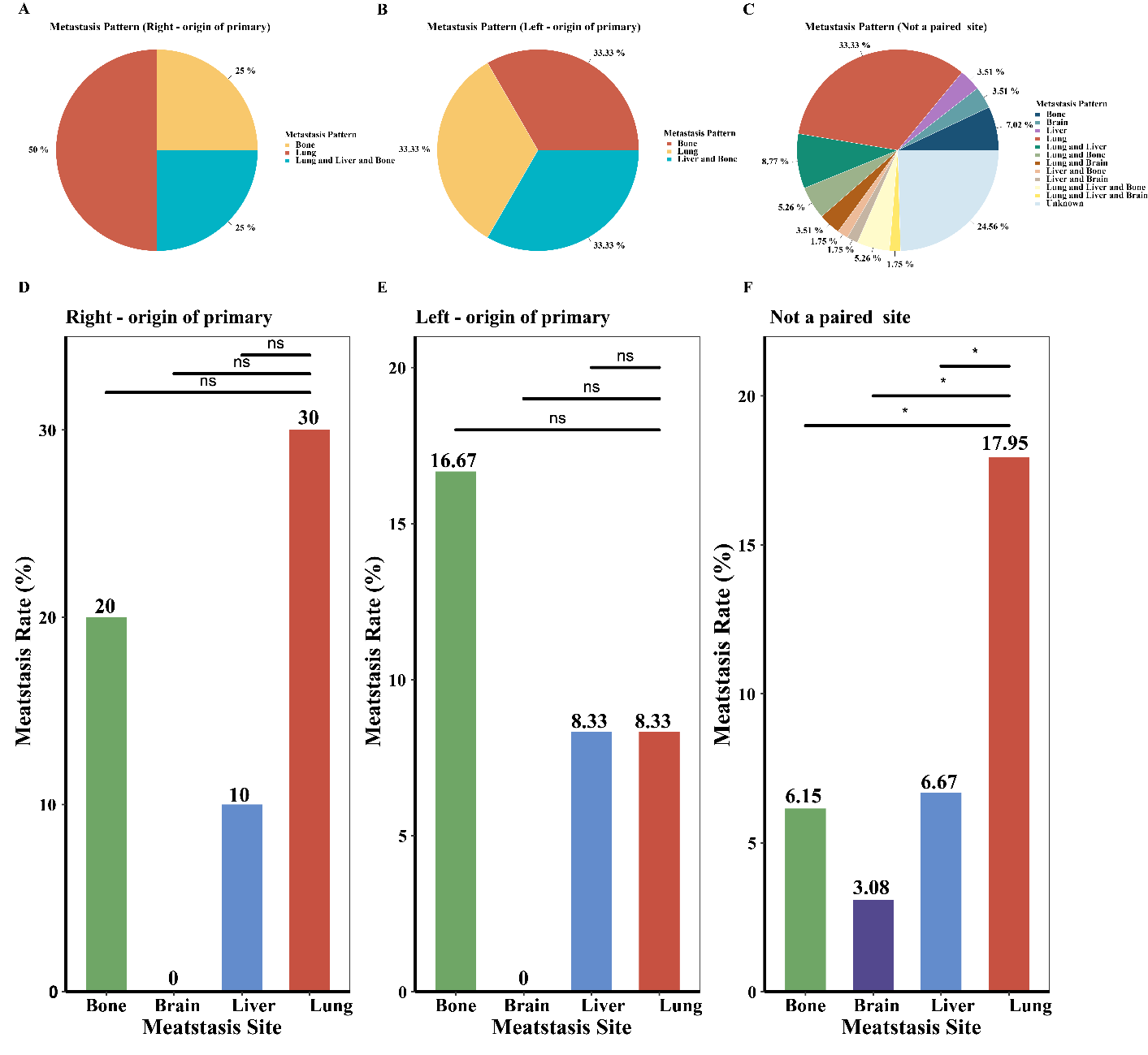


**Supplementary Figure S2.** Proportion and incidence of Distant Metastasis of Different Metastatic Sites in Laterality subgroups. A-C: the proportion of distant metastases; D-F: the incidence of distant metastases.


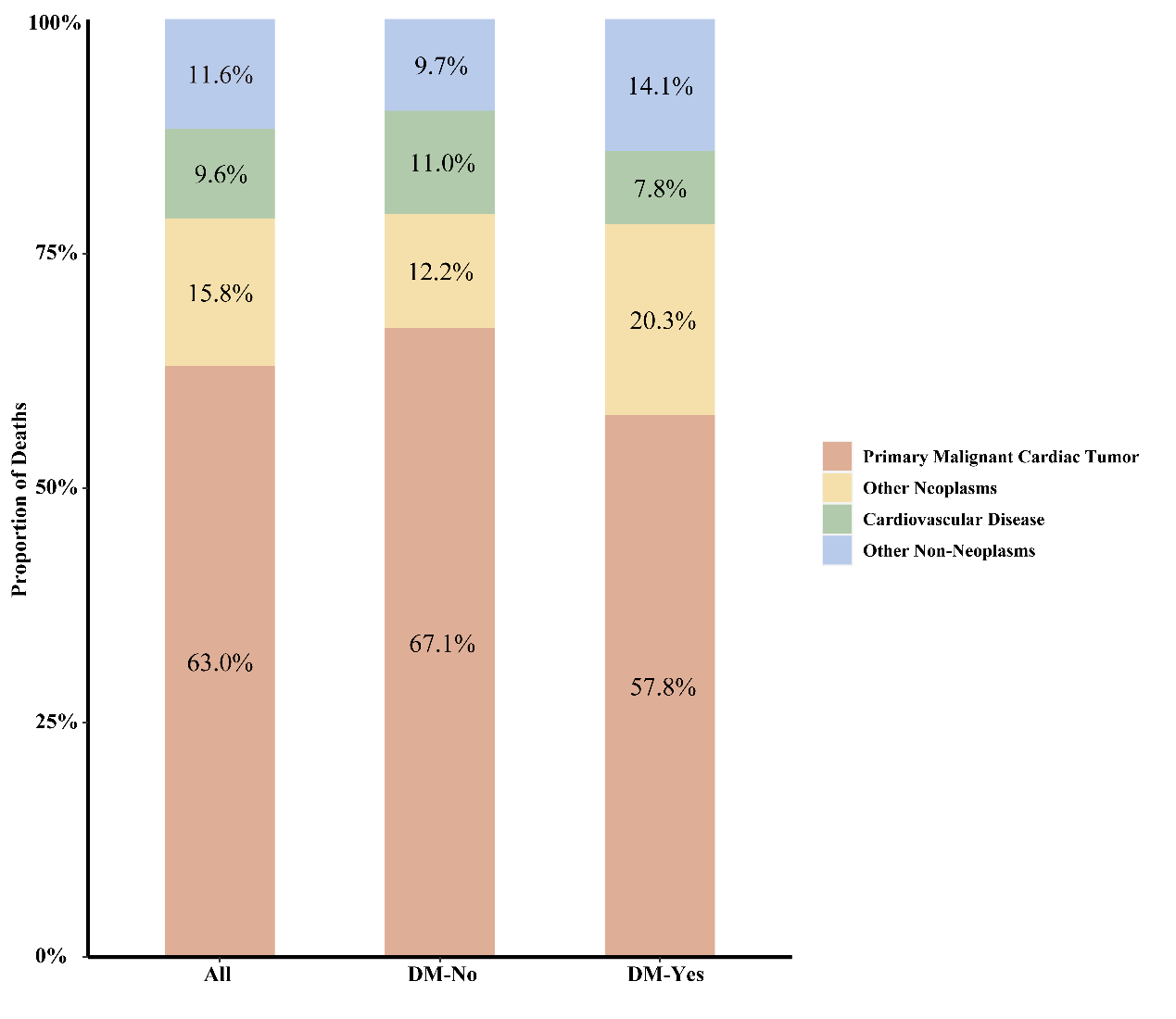


**Supplementary Figure S3.** The Proportion of Death in Patients with Primary Malignant Cardiac Tumors

DM= Distant Metastasis


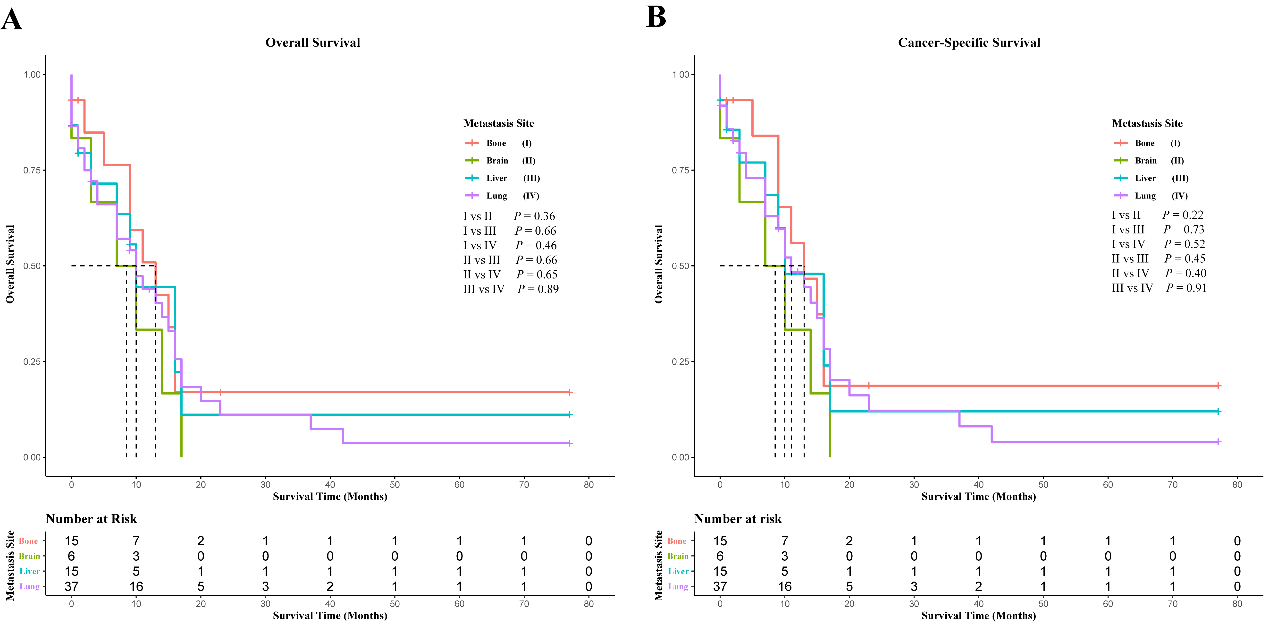


**Supplementary Figure S4.** The Prognostic Impacts of Metastasis Site.

**Supplementary Table S1** Cox Regression Analyses of Prognostic Factors for OS in Primary Malignant Cardiac Tumors

| **Variable** | **Univariate Analysis** | | **Multivariate Analysis^#^** | |
| --- | --- | --- | --- | --- |
|  | **HR (95% CI)** | ***P* value** | **HR (95% CI)** | ***P* value** |
| Distant Metastasis |  |  |  |  |
| No | Reference | | Reference | |
| Yes | 1.72 (1.23-2.39) | 0.001 | 1.47 (1.05-2.07) | 0.025 |
| Age at Diagnosis |  |  |  |  |
| ≤65 years | Reference | | - | - |
| >65 years | 1.16 (0.82-1.65) | 0.39 | - | - |
| Median Household Income |  |  |  |  |
| US $35,000-$49,999 | Reference | | Reference | |
| US $50,000-$74,999 | 0.66 (0.42-1.02) | 0.064 | 0.79 (0.51-1.24) | 0.30 |
| ≥US $75,000 | 0.56 (0.34-0.92) | 0.022 | 0.67 (0.40-1.12) | 0.13 |
| Sex |  |  |  |  |
| Male | Reference | | - | - |
| Female | 0.94 (0.68-1.30) | 0.71 | - | - |
| Year of Diagnosis |  |  |  |  |
| 2010-2013 | Reference | | - | - |
| 2014-2017 | 0.95 (0.67-1.33) | 0.74 | - | - |
| Race |  | 0.67 |  |  |
| White | Reference | | - | - |
| Black | 1.02 (0.67-1.66) | 0.93 | - | - |
| Others* | 0.79 (0.47-1.34) | 0.38 | - | - |
| Grade |  |  |  |  |
| Low | Reference | | - | - |
| High | 1.57 (0.57-4.31) | 0.38 | - | - |
| Others† | 0.62 (0.22-1.79) | 0.38 | - | - |
| Unknown | 1.33 (0.45-3.71) | 0.58 | - | - |
| Histopathology |  |  |  |  |
| Lymphoma | Reference | | Reference | |
| Sarcoma | 2.32 (1.52-3.54) | <0.001 | 2.13 (1.38-3.29) | 0.001 |
| Mesothelioma and Others | 3.84 (1.97-7.48) | <0.001 | 3.52 (1.79-6.92) | <0.001 |
| Size |  |  |  |  |
| ≤4cm | Reference | | - | - |
| >4cm | 1.14 (0.69-1.94) | 0.63 | - | - |
| Unknown | 0.83 (0.48-1.42) | 0.49 | - | - |
| Surgery |  | 0.63 |  |  |
| No | Reference | | - | - |
| Yes | 0.85 (0.62-1.18) | 0.34 | - | - |
| Unknown | - | - | - | - |

^#^Multivariate analysis adjusted for statistically significant factors according to univariate analysis (median household income and histopathology)

*Others races include American Indian/Alaska Native, Asian/Pacific Islander.

†Others includes B-cell, pre-B, B-precursor and B-cell.

OS = overall survival; HR =hazard ratio; CI = confidence interval.

**Supplementary Table S2** Cox Regression Analyses of Prognostic Factors for CSS in Primary Malignant Cardiac Tumors

| **Variable** | **Univariate Analysis** | | **Multivariate Analysis^#^** | | |
| --- | --- | --- | --- | --- | --- |
|  | **HR (95% CI)** | ***P* value** | **HR (95% CI)** | | ***P* value** |
| Distant Metastasis |  |  |  | |  |
| No | Reference | | Reference | | |
| Yes | 1.78 (1.25-2.55) | 0.002 | 1.47 (1.02-2.13) | | 0.039 |
| Age at Diagnosis |  |  |  | |  |
| ≤65 years | Reference | | - | - | |
| >65 years | 0.98 (0.66-1.45) | 0.91 | - | - | |
| Median Household Income |  |  |  | |  |
| US $35,000-$49,999 | Reference | | Reference | | |
| US $50,000-$74,999 | 0.63 (0.39-1.01) | 0.055 | 0.77 (0.48-1.25) | | 0.29 |
| ≥US $75,000 | 0.51 (0.29-0.87) | 0.014 | 0.63 (0.36-1.09) | | 0.10 |
| Sex |  |  |  | |  |
| Male | Reference | | - | - | |
| Female | 0.94 (0.66-1.33) | 0.71 | - | - | |
| Year of Diagnosis |  |  |  |  | |
| 2010-2013 | Reference | | - | - | |
| 2014-2017 | 0.94 (0.65-1.36) | 0.75 | - | - | |
| Race |  | 0.90 |  |  | |
| White | Reference | | - | - | |
| Black | 1.12 (0.66-1.87) | 0.68 | - | - | |
| Others* | 0.97 (0.57-1.65) | 0.90 | - | - | |
| Grade |  |  |  |  | |
| Low | Reference | | - | - | |
| High | 1.45 (0.53-4.00) | 0.47 | - | - | |
| Others† | 0.47 (0.16-1.39) | 0.17 | - | - | |
| Unknown | 1.14 (0.40-3.20) | 0.81 | - | - | |
| Histopathology |  |  |  | |  |
| Lymphoma | Reference | | Reference | | |
| Sarcoma | 2.89 (1.77-4.69) | <0.001 | 2.64 (1.61-4.34) | | <0.001 |
| Mesothelioma and Others | 4.58 (2.18-9.63) | <0.001 | 4.19 (1.97-8.91) | | <0.001 |
| Size |  |  |  |  | |
| ≤4cm | Reference | | - | - | |
| >4cm | 1.03 (0.60-1.77) | 0.92 | - | - | |
| Unknown | 0.64 (0.36-1.12) | 0.12 | - | - | |
| Surgery |  | 0.66 |  |  | |
| No | Reference | | - | - | |
| Yes | 0.85 (0.60-1.21) | 0.37 | - | - | |
| Unknown | 0 (0-4.92E+201) | 0.96 | - | - | |

^#^Multivariate analysis adjusted for statistically significant factors according to univariate analysis (median household income and histopathology)

*Others races include American Indian/Alaska Native, Asian/Pacific Islander.

†Others includes B-cell, pre-B, B-precursor and B-cell.

CSS = cancer specific survival; HR =hazard ratio; CI = confidence interval.
